# Doctors in Ancient Ireland

**Published:** 1904-10-15

**Authors:** 


### Doctors in Ancient Ireland.

Dr. P. W. Joyce, in his recently published "Social History of Ancient Ireland," gives a most interesting account of medical doctors in that country. The profession of medicine was to a great extent hereditary in certain families, and the same person, after an apprenticeship, commonly practised both as a physician and as a surgeon. The ancient Irish name for a physician is liaig (leea), which is radically the same as the old English word *leech*. The medicine-bag carried by a physician was called lés (lace), and without it the leech was assumed to be quite helpless. From the earliest times the Kings and great Irish families had physicians attached to their households, and in the tenth century the physicians, like the rest of the community, took family names. The O'Lees were physicians to the O'Flahertys of Connaught, and the O'Hickeys to the O'Briens of Thomond, to the O'Kennedys of Ormond, and to the Macnamaras of Clare; and the O'Lees and O'Hickeys were of such remote antiquity that they took their names from ancestors who must have sprung into celebrity on account of their skill in medicine, so much so that their ordinary names were changed to ícidhe (eekee), the "healer," and *liaig* (leea), the "leech," and O'Lee signifies the descendant of the leech, and O'Hickey, of the healer. Their profession, like that of other medical families was transmitted from father to son for hundreds of years till it finally died out in times comparatively recent; a good example of the extraordinary tenacity with which families cling to hereditary office in Ireland.

The amount of remuneration of a family leech depended on his own eminence and on the status of the king or chief in whose household he lived. The stipend usually consisted of a tract of land and a residence in the neighbourhood, held free of all tithe and tribute, together with certain allowances and perquisites, and the physician might practise for fee outside his patron's household. Five hundred acres was a usual allowance and some of these estates—now ordinary town lands—retain the family name to this day. Thus Ferrancassidy in Fermanagh was the ferann or land of the O'Cassidys who were hereditary physicians to the Maguires of Fermanagh. The household physician to a king held a very dignified position, and, indeed, lived like a prince, with a household and dependents of his own. He was always among the king's immediate retinue and was entitled to a distinguished place at table.

A leech who, through carelessness, neglect, or gross want of skill, failed to cure a wound, had to pay the same fine to a patient as if he had inflicted the wound with his own hand.

When a physician treated a wound, a certain time was allowed to test whether he had made a good cure. If it broke out afresh before the end of the testing time, the cure was regarded as unsuccessful, and the leech had to return the fee and pay the usual fine. Moreover, if during treatment he and his pupil had lived in the patient's house, he had to refund the cost of maintenance. But he might provide against these penalties by first obtaining a guarantee of immunity. The testing-time for a wound of the hand or arm was a year, for a wound of the leg a little more; for a wound of the head, probably a fracture of the skull, three years. After the testing time the physician and the wounded were both exempt, no matter what happened.

Those who had gone through the prescribed course of study and training were technically and legally qualified physicians. But a person might also set up as a leech and practise without any regular qualification—an "unlawful physician" as he was called, meaning not legally recognised. There was no law to prevent this; but such persons were subject to certain disabilities and dangers not incurred by the regular practitioner. A qualified physician performing a serious surgical operation, had previously to get a guarantee of immunity; if he neglected this he was liable to be cast in the usual damages in case of failure—taking the element of time into consideration. The unqualified practitioner had, of course, to take the same precautions or abide the consequences: and *he should also give notice that he was not a regular physician*, say the Brehon laws. In smaller operations the regular physician was free from liability without any guarantee; while, if there were no guarantee, the other was liable if unsuccessful. It is worthy of remark that in the legendary history of Ireland female physicians are often mentioned.

